# 1,4-Bis(2-pyridylmethyl­eneamino­meth­yl)benzene

**DOI:** 10.1107/S1600536809000646

**Published:** 2009-01-10

**Authors:** Chao Li, Fu-An Sun, Ming-Yang He, Huan Xu, Qun Chen

**Affiliations:** a Key Laboratory of Fine Petro-Chemical technology, Jiangsu Polytechnic University, Changzhou 213164, People’s Republic of China

## Abstract

The asymmetric unit of the centrosymmetric title compound, C_20_H_18_N_4_, contains one half-mol­ecule. The pyridine and benzene rings are oriented at a dihedral angle of 77.21 (7)°.

## Related literature

For general background, see: Barboiu *et al.* (2006 ▶); Keegan *et al.* (2002 ▶); Yue *et al.* (2004 ▶). For bond-length data, see: Allen *et al.* (1987 ▶).
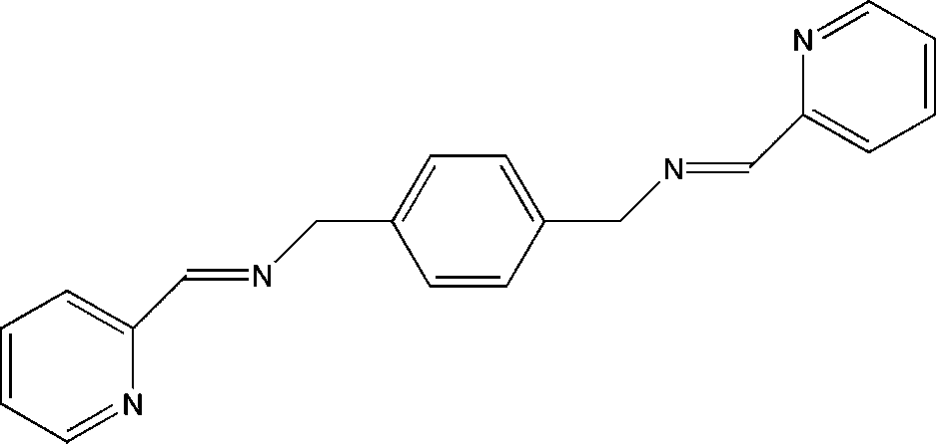


## Experimental

### 

#### Crystal data

C_20_H_18_N_4_
*M* *_r_* = 314.38
Triclinic, 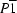

*a* = 4.527 (3) Å
*b* = 10.117 (6) Å
*c* = 10.456 (6) Å
α = 61.086 (7)°
β = 88.543 (8)°
γ = 82.242 (8)°
*V* = 414.9 (4) Å^3^
*Z* = 1
Mo *K*α radiation
μ = 0.08 mm^−1^
*T* = 296 (2) K
0.32 × 0.30 × 0.23 mm

#### Data collection

Bruker SMART CCD area-detector diffractometer
Absorption correction: multi-scan (*SADABS*; Sheldrick, 2000 ▶) *T*_min_ = 0.976, *T*_max_ = 0.990
2909 measured reflections
1449 independent reflections
1250 reflections with *I* > 2σ(*I*)
*R*_int_ = 0.021

#### Refinement

*R*[*F* ^2^ > 2σ(*F* ^2^)] = 0.036
*wR*(*F* ^2^) = 0.105
*S* = 1.01
1449 reflections
109 parameters
H-atom parameters constrained
Δρ_max_ = 0.14 e Å^−3^
Δρ_min_ = −0.18 e Å^−3^

### 

Data collection: *SMART* (Bruker, 2000 ▶); cell refinement: *SAINT* (Bruker, 2000 ▶); data reduction: *SAINT*; program(s) used to solve structure: *SHELXTL* (Sheldrick, 2008 ▶); program(s) used to refine structure: *SHELXTL*; molecular graphics: *SHELXTL*; software used to prepare material for publication: *SHELXTL*.

## Supplementary Material

Crystal structure: contains datablocks I, global. DOI: 10.1107/S1600536809000646/hk2592sup1.cif

Structure factors: contains datablocks I. DOI: 10.1107/S1600536809000646/hk2592Isup2.hkl

Additional supplementary materials:  crystallographic information; 3D view; checkCIF report

## Figures and Tables

**Fig. 1.**
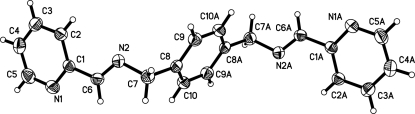
The molecular structure of the title molecule, with the atom-numbering scheme. Displacement ellipsoids are drawn at the 30% probability level [symmetry code: (A) 1 - x, 1 - y, 1 - z].

## supplementary crystallographic information

### Comment

Bipyridyl-type bidentate Schiff base ligands have been utilized intensively to assemble various coordination polymers with interesting topologies and fascinating structural diversities (Barboiu *et al.*,  2006; Keegan *et al.*,  2002; Yue *et al.*,  2004). We report herein the crystal structure of the title compound.

The asymmetric unit of the title compound (Fig. 1) contains one-half of the centrosymmetric molecule, where the bond lengths (Allen *et al.*, 1987) and angles are within normal ranges. Rings A (N1/C1-C5) and B (C8-C10/C8A-C10A) are, of course, planar, and they are oriented at a dihedral angle of 77.21 (7)° [symmetry code: (A) 1 - x, 1 - y, 1 - z].

### Experimental

The title compound was prepared from the condensation reaction between pyridine-2-carboxaldehyde (100 mmol) and 1,4-benzenedimethanamine (50 mmol) in tetrahydrofuran (yield; 83%). Crystals suitable for X-ray analysis were obtained by slow evaporation of a methanol solution at room temperature.

### Refinement

H atoms were positioned geometrically, with C-H = 0.93 and 0.97 Å for aromatic and methylene H, respectively, and constrained to ride on their parent atoms, with U_iso_(H) = 1.2U_eq_(C).

### Crystal data

**Table:**

C_20_H_18_N_4_	*Z* = 1
*M**_r_* = 314.38	*F*(000) = 166
Triclinic, *P*1	*D*_x_ = 1.258 Mg m^−^^3^
Hall symbol: -P 1	Mo *K*α radiation, λ = 0.71073 Å
*a* = 4.527 (3) Å	Cell parameters from 1582 reflections
*b* = 10.117 (6) Å	θ = 2.2–27.0°
*c* = 10.456 (6) Å	µ = 0.08 mm^−^^1^
α = 61.086 (7)°	*T* = 296 K
β = 88.543 (8)°	Block, colorless
γ = 82.242 (8)°	0.32 × 0.30 × 0.23 mm
*V* = 414.9 (4) Å^3^	

### Data collection

**Table:**

Bruker SMART CCD area-detector diffractometer	1449 independent reflections
Radiation source: fine-focus sealed tube	1250 reflections with *I* > 2σ(*I*)
graphite	*R*_int_ = 0.021
φ and ω scans	θ_max_ = 25.0°, θ_min_ = 2.2°
Absorption correction: multi-scan (*SADABS*; Sheldrick, 2000)	*h* = −5→5
*T*_min_ = 0.976, *T*_max_ = 0.990	*k* = −12→12
2909 measured reflections	*l* = −12→11

### Refinement

**Table:**

Refinement on *F*^2^	Primary atom site location: structure-invariant direct methods
Least-squares matrix: full	Secondary atom site location: difference Fourier map
*R*[*F*^2^ > 2σ(*F*^2^)] = 0.036	Hydrogen site location: inferred from neighbouring sites
*wR*(*F*^2^) = 0.105	H-atom parameters constrained
*S* = 1.01	*w* = 1/[σ^2^(*F*_o_^2^) + (0.0582*P*)^2^ + 0.0501*P*] where *P* = (*F*_o_^2^ + 2*F*_c_^2^)/3
1449 reflections	(Δ/σ)_max_ < 0.001
109 parameters	Δρ_max_ = 0.14 e Å^−^^3^
0 restraints	Δρ_min_ = −0.17 e Å^−^^3^

### Special details

**Table:**

Geometry. All e.s.d.'s (except the e.s.d. in the dihedral angle between two l.s. planes) are estimated using the full covariance matrix. The cell e.s.d.'s are taken into account individually in the estimation of e.s.d.'s in distances, angles and torsion angles; correlations between e.s.d.'s in cell parameters are only used when they are defined by crystal symmetry. An approximate (isotropic) treatment of cell e.s.d.'s is used for estimating e.s.d.'s involving l.s. planes.
Refinement. Refinement of *F*^2^ against ALL reflections. The weighted *R*-factor *wR* and goodness of fit *S* are based on *F*^2^, conventional *R*-factors *R* are based on *F*, with *F* set to zero for negative *F*^2^. The threshold expression of *F*^2^ > σ(*F*^2^) is used only for calculating *R*-factors(gt) *etc*. and is not relevant to the choice of reflections for refinement. *R*-factors based on *F*^2^ are statistically about twice as large as those based on *F*, and *R*- factors based on ALL data will be even larger.

### Fractional atomic coordinates and isotropic or equivalent isotropic displacement parameters (Å^2^)

**Table:**

	*x*	*y*	*z*	*U*_iso_*/*U*_eq_	
N1	0.8015 (3)	0.26368 (14)	1.18773 (13)	0.0628 (3)	
N2	0.4321 (2)	0.26607 (12)	0.89307 (11)	0.0516 (3)	
C1	0.7265 (3)	0.20335 (14)	1.10637 (13)	0.0477 (3)	
C2	0.8567 (3)	0.06149 (15)	1.12895 (14)	0.0536 (3)	
H2	0.7948	0.0215	1.0722	0.064*	
C3	1.0786 (3)	−0.01952 (17)	1.23630 (16)	0.0622 (4)	
H3	1.1727	−0.1140	1.2519	0.075*	
C4	1.1584 (4)	0.04142 (19)	1.31980 (16)	0.0673 (4)	
H4	1.3071	−0.0110	1.3937	0.081*	
C5	1.0143 (4)	0.18146 (19)	1.29230 (17)	0.0714 (5)	
H5	1.0687	0.2215	1.3503	0.086*	
C6	0.4970 (3)	0.29926 (14)	0.98916 (14)	0.0497 (3)	
H6	0.3968	0.3873	0.9862	0.060*	
C7	0.2103 (3)	0.37330 (16)	0.77804 (14)	0.0560 (4)	
H7A	0.1281	0.4543	0.7986	0.067*	
H7B	0.0484	0.3210	0.7748	0.067*	
C8	0.3559 (3)	0.43975 (14)	0.63283 (13)	0.0470 (3)	
C9	0.3629 (3)	0.37288 (15)	0.54431 (14)	0.0541 (3)	
H9	0.2705	0.2866	0.5733	0.065*	
C10	0.4953 (3)	0.56793 (15)	0.58621 (14)	0.0544 (4)	
H10	0.4935	0.6150	0.6438	0.065*	

### Atomic displacement parameters (Å^2^)

**Table:**

	*U*^11^	*U*^22^	*U*^33^	*U*^12^	*U*^13^	*U*^23^
N1	0.0703 (8)	0.0659 (7)	0.0620 (7)	−0.0068 (6)	−0.0016 (6)	−0.0391 (6)
N2	0.0562 (7)	0.0502 (6)	0.0452 (6)	−0.0045 (5)	0.0029 (5)	−0.0215 (5)
C1	0.0499 (7)	0.0519 (7)	0.0441 (7)	−0.0121 (6)	0.0107 (5)	−0.0246 (6)
C2	0.0571 (8)	0.0527 (7)	0.0524 (7)	−0.0080 (6)	0.0060 (6)	−0.0268 (6)
C3	0.0614 (9)	0.0552 (8)	0.0598 (8)	−0.0025 (6)	0.0032 (7)	−0.0214 (7)
C4	0.0614 (9)	0.0776 (10)	0.0520 (8)	−0.0078 (8)	−0.0027 (7)	−0.0231 (8)
C5	0.0784 (11)	0.0846 (11)	0.0626 (9)	−0.0120 (9)	−0.0046 (8)	−0.0441 (9)
C6	0.0521 (7)	0.0461 (7)	0.0512 (7)	−0.0057 (5)	0.0079 (6)	−0.0245 (6)
C7	0.0507 (8)	0.0607 (8)	0.0542 (8)	−0.0029 (6)	0.0009 (6)	−0.0271 (7)
C8	0.0393 (6)	0.0488 (7)	0.0470 (7)	0.0033 (5)	−0.0071 (5)	−0.0206 (6)
C9	0.0563 (8)	0.0498 (7)	0.0571 (8)	−0.0099 (6)	−0.0004 (6)	−0.0258 (6)
C10	0.0593 (8)	0.0566 (8)	0.0539 (8)	−0.0048 (6)	−0.0020 (6)	−0.0326 (6)

### Geometric parameters (Å, °)

**Table:**

N1—C1	1.3358 (17)	C6—C1	1.472 (2)
N1—C5	1.332 (2)	C6—H6	0.9300
N2—C6	1.2555 (17)	C7—H7A	0.9700
N2—C7	1.4620 (18)	C7—H7B	0.9700
C1—C2	1.383 (2)	C8—C7	1.5078 (19)
C2—C3	1.373 (2)	C8—C9	1.3832 (19)
C2—H2	0.9300	C8—C10	1.383 (2)
C3—C4	1.367 (2)	C9—C10^i^	1.379 (2)
C3—H3	0.9300	C9—H9	0.9300
C4—C5	1.372 (2)	C10—C9^i^	1.379 (2)
C4—H4	0.9300	C10—H10	0.9300
C5—H5	0.9300		
			
C5—N1—C1	116.79 (13)	N2—C6—H6	118.9
C6—N2—C7	117.39 (12)	C1—C6—H6	118.9
N1—C1—C2	122.69 (13)	N2—C7—C8	109.34 (11)
N1—C1—C6	115.30 (12)	N2—C7—H7A	109.8
C2—C1—C6	122.01 (12)	C8—C7—H7A	109.8
C3—C2—C1	119.09 (13)	N2—C7—H7B	109.8
C3—C2—H2	120.5	C8—C7—H7B	109.8
C1—C2—H2	120.5	H7A—C7—H7B	108.3
C4—C3—C2	118.73 (14)	C9—C8—C7	121.61 (12)
C4—C3—H3	120.6	C10—C8—C7	120.48 (11)
C2—C3—H3	120.6	C10^i^—C9—C8	121.07 (13)
C3—C4—C5	118.57 (14)	C10—C8—C9	117.88 (12)
C3—C4—H4	120.7	C10^i^—C9—H9	119.5
C5—C4—H4	120.7	C8—C9—H9	119.5
N1—C5—C4	124.10 (14)	C9^i^—C10—C8	121.05 (12)
N1—C5—H5	117.9	C9^i^—C10—H10	119.5
C4—C5—H5	117.9	C8—C10—H10	119.5
N2—C6—C1	122.13 (12)		
			
C5—N1—C1—C2	1.0 (2)	C3—C4—C5—N1	−0.7 (2)
C5—N1—C1—C6	−178.28 (12)	N2—C6—C1—N1	170.79 (11)
C1—N1—C5—C4	0.3 (2)	N2—C6—C1—C2	−8.52 (19)
C7—N2—C6—C1	−177.26 (11)	C9—C8—C7—N2	90.82 (14)
C6—N2—C7—C8	115.21 (13)	C10—C8—C7—N2	−87.19 (15)
N1—C1—C2—C3	−2.0 (2)	C7—C8—C9—C10^i^	−178.00 (12)
C6—C1—C2—C3	177.27 (11)	C10—C8—C9—C10^i^	0.1 (2)
C1—C2—C3—C4	1.6 (2)	C7—C8—C10—C9^i^	178.03 (12)
C2—C3—C4—C5	−0.3 (2)	C9—C8—C10—C9^i^	−0.1 (2)

Symmetry codes: (i) −*x*+1, −*y*+1, −*z*+1.
